# The emerging role of Panx1 as a potential therapeutic target for chronic pain

**DOI:** 10.1186/s40779-024-00549-0

**Published:** 2024-07-05

**Authors:** Mabel L. Cummins, Skylar Wechsler, Grace Delmonte, Joseph J. Schlesinger

**Affiliations:** 1 Department of Anesthesiology, Vanderbilt University Medical Center, Nashville, TN 37212 USA; 2 Department of Anesthesiology, Division of Critical Care Medicine, Vanderbilt University Medical Center, Nashville, TN 37212 USA

**Keywords:** Panx1, Chronic pain, Neuropathic pain, Inflammation, Animal model, Translation

Chronic pain is a leading cause of disability and affects over 30% of military veterans and active duty personnel [1]. Pharmacological approaches to long-term chronic pain management increase the risk of negative effects such as addiction, cardiovascular complications, and immunosuppression [2]. In contrast, non-drug treatments like audio analgesia have demonstrated efficacy in pain relief without these side effects. Given the prevalence of chronic pain in the military population, it is essential to explore new treatments that effectively target its underlying mechanisms. One promising option is the Pannexin 1 (Panx1) channel-forming protein. Panx1 is a large-pore ion channel in the plasma membrane of dorsal root ganglion (DRG) neurons that allows for the release of Adenosine triphosphate (ATP). This commentary elaborates on the findings of Xing et al. [3], who investigated the role of Panx1 in peripheral pain sensitization following inflammatory stimuli. We examine the role of Panx1 in chronic pain, critique Xing et al.’s [3] adjuvant selection and power analysis, and discuss the challenges of translating findings from animal models to human conditions.

Pain is often caused by tissue damage or other chemical stimuli that induce the release of inflammatory mediators (e.g., histamines), which stimulate afferent fibers and contribute to the sensation of pain [4]. In the acute stages of pain, inflammation is beneficial as it elicits an immune response. However, chronic inflammation leads to long-lasting pain by increasing the expression of ligand and voltage-gated ion channels, resulting in hyperexcitability of DRG neurons [4]. The conduction of ATP through Panx1 channels is essential for the development of inflammatory pain because it mediates the communication between DRG neurons [5, 6]. Previous work has shown that inhibition of Panx1 decreases pain sensitivity induced by inflammatory stimuli [7], revealing a potential therapeutic target for inflammatory pain. Current research also suggests that the Wnt/β-catenin signaling pathway, which regulates neuronal-related gene expression and cell proliferation [8], is upregulated in neuropathic pain [9]. Notably, Xing et al. [3] determined that the Wnt/β-catenin pathway may modulate the activity of Panx1, so targeting the interaction of these elements is an attractive strategy for the treatment of pain.

In their study, Xing et al. [3] used complete Freund’s adjuvant (CFA), an inflammatory agent consisting of heat-killed mycobacteria, to induce hypersensitivity in the mouse hind paw. While CFA is an effective immune stimulator in animals, its clinical applicability is limited because it cannot be used in humans to induce chronic pain due to its potency. Furthermore, the continual pain induced by CFA can interfere with behavioral assessments by reducing exploratory behavior and potentially increasing immobility in certain tests. Though CFA has been widely used in many studies due to its efficacy, researchers should consider the specific requirements of their model, such as persistence or chronicity of pain, which can be induced by various adjuvants like incomplete Freund’s adjuvant, carrageenan, formalin, or antigens in presensitized rodents.

Additionally, the methodology and lack of transparency in this paper need to be critiqued to promote more robust future studies, thereby increasing the translational quality of animal studies [10]. This study lacked a calculation of statistical power, and the cited study did not relate to a power analysis recommendation. And there was no report on the calculation and justification of various sample sizes used in this study. Also, the study did not mention the use of bias-reducing measures such as random assignment or blinding during the von Frey behavior assessment. Due to the potential influence of multiple factors on animal studies, methodological transparency is key to ensuring the robustness and replicability of studies.

Finally, Xing et al. [3] employed the von Frey test to assess allodynia, a behavioral measure of pain avoidance, in conjunction with electrophysiology to quantify the neuronal excitability of the *Panx1* mutants. The utilization of two complementary methods strengthens the validity of their results, but this study still faces the challenge of demonstrating the applicability of these findings to humans. Animal models for induced neuropathic pain have limited predictive validity when investigating pharmacological treatments, as they do not fully replicate the etiology of the human condition [10]. Furthermore, using young and healthy for chronic pain studies contrasts with the patient populations that typically develop chronic neuropathic pain (usually comorbid with disease), reducing the true predictivity of these models [10]. For future research, it is important to consider human pain conditions (e.g., injury or disease-related pain) and utilize them to guide which of the different mechanisms for inducing pain in rodents is the most appropriate.

Chronic pain has a significant impact on service members, necessitating the military medical system to find effective treatments to boost their readiness for duty and improve their quality of life. Due to the challenges in investigating treatments for chronic pain in human subjects, additional efforts are needed to adapt animal models to the human condition and ensure high-quality, reproducible studies. Xing et al. [3] provide promising insights into how Panx1 facilitates inflammatory pain through experiments on pain sensitivity and neuronal excitability. Few studies have demonstrated the translational progress of Panx1 as the target for inflammation and disease control [11–13], so further research characterizing the interactions of pharmacological treatments on Panx1, the Wnt/β-catenin signaling pathway, and DRG may elucidate promising avenues for the treatment of chronic pain in the military population.

## Data Availability

Not applicable.

## Abbreviations

ATP: Adenosine triphosphate
CFA: Complete Freund’s adjuvant
DRG: Dorsal root ganglion
Panx1: Pannexin1
